# Effect of home-based exercise prehabilitation on postoperative outcomes in colorectal cancer surgery: a systematic review and meta-analysis

**DOI:** 10.1007/s00520-024-09069-y

**Published:** 2024-12-12

**Authors:** Pedro Machado, André Paixão, Bárbara Oliveiros, Raul A. Martins, Joana Cruz

**Affiliations:** 1 School of Health Sciences of the Polytechnic of Leiria (ESSLei), Center for Innovative Care and Health Technology (ciTechCare), Leiria, Portugal; 2 Research Unit for Sport and Physical Activity (CIDAF, UID/PTD/04213/2019), Faculty of Sport Sciences and Physical Education, University of Coimbra, Coimbra, Portugal; 3 Physical Therapy Clinics, Physioclem, Alcobaça, Portugal; 4 Sport Sciences School of Rio Maior (ESDRM), Santarém Polytechnic University, Santarém, Portugal; 5 Laboratory of Biostatistics and Medical Informatics (LBIM), Faculty of Medicine, University of Coimbra, Coimbra, Portugal; 6 Coimbra Institute for Clinical and Biomedical Research (iCBR), Faculty of Medicine, University of Coimbra, Coimbra, Portugal; 7 Institute for Biomedical Imaging and Translational Research (CIBIT), University of Coimbra, Coimbra, Portugal

**Keywords:** Colorectal cancer, Exercise training, Prehabilitation, Surgical oncology, Perioperative medicine

## Abstract

**Purpose:**

Home-based exercise training may improve access to surgical prehabilitation in colorectal cancer (CRC) patients, but its efficacy remains unclear. This study systematically investigated the effects of home-based exercise prehabilitation on postoperative exercise capacity, complications, length of hospital stay, and health-related quality of life (HRQoL) in CRC patients.

**Methods:**

Randomized controlled trials (RCTs) comparing home-based exercise prehabilitation with control in CRC patients were eligible. We searched MEDLINE, Scopus, Web of Science, PEDro, and SPORTDiscus from their inception to June 3, 2024. Methodological quality was assessed using the PEDro scale, and certainty of evidence was assessed using GRADE. Data were synthesized using random-effects meta-analyses, with sensitivity analysis on studies with good methodological quality (PEDro score ≥ 6).

**Results:**

Eight RCTs involving 1092 participants were included. The primary analysis showed a significant improvement in postoperative 6-min walk distance following home-based exercise prehabilitation compared to control (mean difference (MD) = 30.62: 95% CI: [2.94; 57.79]; low-certainty evidence). However, sensitivity analysis revealed no significant between-group differences (MD = 22.60: 95% CI: [− 6.27; 51.46]). No significant effects of home-based exercise prehabilitation were found on postoperative complications (risk ratio = 1.00: 95% CI: [− 0.78; 1.29]; moderate‐certainty evidence), length of hospital stay (MD = − 0.20: 95% CI: [− 0.65; 0.23]; moderate‐certainty evidence), and HRQoL (physical functioning: MD = 2.62: 95% CI: [− 6.16; 11.39]; mental functioning: MD = 1.35: 95% CI: [− 6.95; 9.65]; low and very-low certainty evidence).

**Conclusion:**

Home-based exercise prehabilitation does not reduce postoperative complications and length of hospital stay after CRC surgery. Its effects on postoperative exercise capacity and HRQoL remain uncertain due to low-quality evidence.

**Supplementary Information:**

The online version contains supplementary material available at 10.1007/s00520-024-09069-y.

## Introduction

Colorectal cancer surgery is a growing challenge, with an estimated increase of 54–62% in the global number of surgical candidates by 2040 [48]. This represents over 900,000 patients requiring surgical treatment worldwide [48].

Despite substantial advancements in colorectal cancer surgery, an overall postoperative complication rate of 30–40% has been reported [31, 62], highlighting the high-risk nature of this procedure. Postoperative complications are associated with delayed administration of adjuvant chemotherapy, which may result in worse disease-free and overall survival [30, 56].

Colorectal cancer surgery also has a detrimental impact on patients’ health-related quality of life (HRQoL), leading to functional limitations and an increase in symptoms of pain, fatigue, and dyspnea [2, 54, 59, 60, 64]. Moreover, despite the general tendency to improvement with follow-up, approximately 40% of patients have worse quality of life 6 months after surgery, and around one-third do not regain their preoperative levels 5 years after treatment [67].

Prehabilitation regimens involving an exercise training component (exercise-based prehabilitation) have been advocated to enhance recovery after colorectal cancer surgery, based on the rationale that they may optimize physiological reserve, thereby improving patients’ resilience to withstand the stress of tumor resection [39, 66].

A critical factor in optimizing the effectiveness of exercise-based prehabilitation and implementing this intervention as part of routine perioperative care is to maximize patients’ adherence [20]. This is particularly relevant in the preoperative period due to the time constraints imposed by the surgical schedule for implementing exercise training programs [20]. Therefore, the context where prehabilitation is delivered should be well-considered when designing this type of intervention [20, 65].

In patients scheduled for colorectal cancer surgery, exercise-based prehabilitation has been delivered at the hospital [36, 66], in the community [6, 7], and home-based environments [13, 14, 45]. While home-based exercise programs can improve access to prehabilitation by overcoming environmental barriers, such as transportation problems [20, 44, 63], their efficacy on postoperative outcomes is not well established. A growing body of evidence has shown that exercise-based prehabilitation improves exercise capacity and may reduce complications after colorectal surgery [19, 23]; however, these beneficial effects result mostly from programs conducted at the hospital [36, 42, 55, 66] and in community-based settings [6, 7], with conflicting evidence provided by clinical trials involving home-based interventions [11, 13, 14, 22, 45, 58]. Therefore, the efficacy of home-based exercise prehabilitation still needs to be examined.

This systematic review aimed to determine the effects of home-based exercise prehabilitation programs on postoperative outcomes in patients undergoing colorectal cancer surgery.

## Material and methods

### Protocol and reporting

This systematic review followed the Preferred Reporting Items for Systematic Reviews and Meta-Analysis (PRISMA) guidelines [46].

The protocol was pre-registered on the International Prospective Register of Systematic Reviews (PROSPERO), registration number CRD42024554360.

### Eligibility criteria

The eligibility criteria were defined using the participants, intervention, comparator, outcome, and type of study (PICOS) approach [51].

#### Type of participants

The population included adult patients (age ≥ 18 years), scheduled to undergo colon or rectal cancer resection. Studies enrolling more than 20% of participants with benign disease or other cancer types were excluded unless a subgroup analysis was available.

#### Type of intervention

Surgical prehabilitation programs involving a home-based exercise training component were included. Exercise training was defined as a type of physical activity that consists of a well-defined and structured plan aiming to increase or maintain the person's physical fitness [1]. Exercise training could be implemented as a single-modality intervention (unimodal prehabilitation), or in combination with additional elements such as nutritional and psychological interventions (multimodal prehabilitation). The training sessions could be supervised or unsupervised, or both, and include aerobic or resistance training, or a combination. Exercise regimens that combined aerobic or resistance training with other training modalities, such as respiratory muscle training or pelvic floor muscle training were included; however, exercise regimens that investigated the effects of respiratory or pelvic muscle training alone were excluded. Interventions that combined home-based and facility-based (e.g., in-hospital, clinic, laboratory) exercise training were excluded if more than 30% of planned training sessions were facility based.

#### Type of comparison

The control group could not have performed any type of structured exercise training before colorectal cancer surgery (only standard preoperative care with no exercise training). Nevertheless, general advice about physical activity, without a structured exercise prescription, was considered as a comparison intervention.

#### Type of outcome

The primary outcome was postoperative exercise capacity, measured using field or laboratory-based tests (e.g., 6-min walk test (6MWT), incremental shuttle walk test, cardiopulmonary exercise test).

The secondary outcomes were:

- postoperative length of hospital stay;
- postoperative complications; and
- HRQoL.

For exercise capacity and HRQoL, the first assessment after surgery was considered. Studies that only reported preoperative values were excluded.

#### Type of studies

Studies were deemed eligible if they were randomized controlled trials (RCTs), published in English, Spanish, or Portuguese until June 3, 2024. The trials had to allocate participants to home-based exercise prehabilitation versus a control group. Conference abstracts and unpublished manuscripts were excluded.

### Information sources

A systematic electronic search was performed in MEDLINE via PubMed, Physiotherapy Evidence Database (PEDro), Scopus, Web of Science, and SPORTDiscus via EBSCO, from inception to June 3, 2024. References from retrieved articles were reviewed for additional studies.

### Search strategy

The search strategy combined Key Medical Subject Headings (MeSH) and free-text words related to “colorectal cancer,” “surgery,” “prehabilitation,” and “exercise training,” using Boolean operators (OR/AND). The full search strategies and filters applied to each bibliographic database are presented in Supplementary Table 1.

### Selection of studies

After deduplication, titles and abstracts were screened by two independent reviewers (PM and AP). If there were doubts about a potential article following the inclusion criteria or if there was incomplete information to make a clear inclusion or exclusion decision, that article was kept for the following phase (analysis of its full text). The second screening phase was also carried out independently by the same reviewers. Studies that were identified by mutual consent were included in the systematic review. In case of disagreement, a third reviewer (JC) was consulted, and the final decision was based on the combination of the three opinions.

Cohen’s kappa coefficient was calculated to evaluate interrater reliability in the initial and full-text screenings [38]. The kappa values can be interpreted as follows: values ≤ 0 indicating no agreement and 0.01–0.20 as none to slight, 0.21–0.40 as fair, 0.41–0.60 as moderate, 0.61–0.80 as substantial, and 0.81–1.00 as almost perfect agreement [38].

### Data extraction

Data extraction was independently performed by two reviewers (PM and AP) with any discrepancies being resolved through discussion with a third reviewer (JC). Relevant extracted data were organized using standardized tables, that included the following topics: (1) study characteristics; (2) participants’ demographic and clinical characteristics; (3) surgical characteristics; (4) training dose; (4) exercise adherence and adverse events; and (5) outcome measurements. When information regarding any of the above topics was unclear or insufficient, the authors of the papers were contacted to provide details.

### Quality assessment

Critical appraisal of the trials’ methodological quality was performed by two reviewers (PM and AP), using the PEDro scale [52]. Disagreements were resolved by consensus, with a third reviewer (JC) acting as a mediator if necessary. The PEDro scale comprises 11 items: eligibility criteria, randomized allocation, hidden allocation, baseline comparison between groups, participants, physiotherapists, and blind assessors, adequate follow-up, intention to treat the analysis, comparison between groups, and point estimate and variability. Based on these items, a score of 0 to 10 is attributed to the RCTs [15]. Authors have suggested that studies with a score of 0 to 3 have a “poor” methodological quality, from 4 to 5 “reasonable,” 6 to 8 “good,” and 9 to 10 “excellent” [15].

### Data synthesis and analysis

Meta-analyses were conducted if data from two or more eligible trials could be combined. Risk ratio (RR) with 95% confidence intervals (CI) was used as a summary measure for postoperative complications, and mean differences (MDs) with 95% CI were used as a summary measure for exercise capacity, length of hospital stay, and HRQoL. A random-effects model was used in the meta-analysis, as it combines sampling error and between-study variance to estimate effect size. Sensitivity analysis was undertaken by pooling the data of studies with good methodological quality (PEDro score ≥ 6).

Inconsistency across studies was assessed using the *I*-squared (*I*^2^) and was interpreted as follows [33]: *I*^2^ = 0–40%: might not be important; *I*^2^ = 50–90%: may represent substantial heterogeneity; *I*^2^ = 75–100%: considerable heterogeneity.

When insufficient data were provided in a study to estimate the exercise training effect, we contacted the authors to provide the required data [mean ($\overline{x }$) and standard deviation (SD)]. When $\overline{x }$ and SD were not reported in a study and not provided by the authors, they were calculated from the median (*m*), first (*Q*_1_), and third quartiles (*Q*_3_), or sample size (*N*) and 95% CI [32, 33, 61]:

$$\overline{x}\approx \frac{{Q }_{1}+m+{Q}_{3}}{3}$$

$$SD = \frac{{Q}_{3}-{Q}_{1}}{1.35}$$

$$D = \sqrt{N}\times \left(upper \;CI-lower \;CI\right)/({t}_{\text{alpha},\text{ df }}\times 2)$$

All statistical analyses were conducted using the statistical software Comprehensive Meta-Analysis (CMA) (Biostat, Englewood, NJ, USA, version 3.3.070) [10]. A *p*-value of < 0.05 was considered statistically significant.

### Publication bias

The publication bias was calculated using the software CMA [10], generating a funnel plot by the standard error (SE) and the standard difference in means to determine whether the plot was balanced. The risk of publication bias was assessed by the visual inspection of the funnel plots and using Egger’s test to provide a more objective and accurate assessment of funnel plot asymmetry than subjective visual assessment [53].

### Certainty of evidence

The Grading of Recommendations Assessment, Development, and Evaluation (GRADE) approach was used to assess the certainty of evidence [29]. Evidence was downgraded if there were issues with risk of bias across studies, inconsistency of results, publication bias, imprecision, and indirectness, according to the recommendations of the GRADE Working Group [24–28].

## Results

### Search results

A total of 2112 records were obtained from electronic databases. After deduplication, 1473 records were screened for content, from which 8 RCTs were included.

**Fig. 1:**
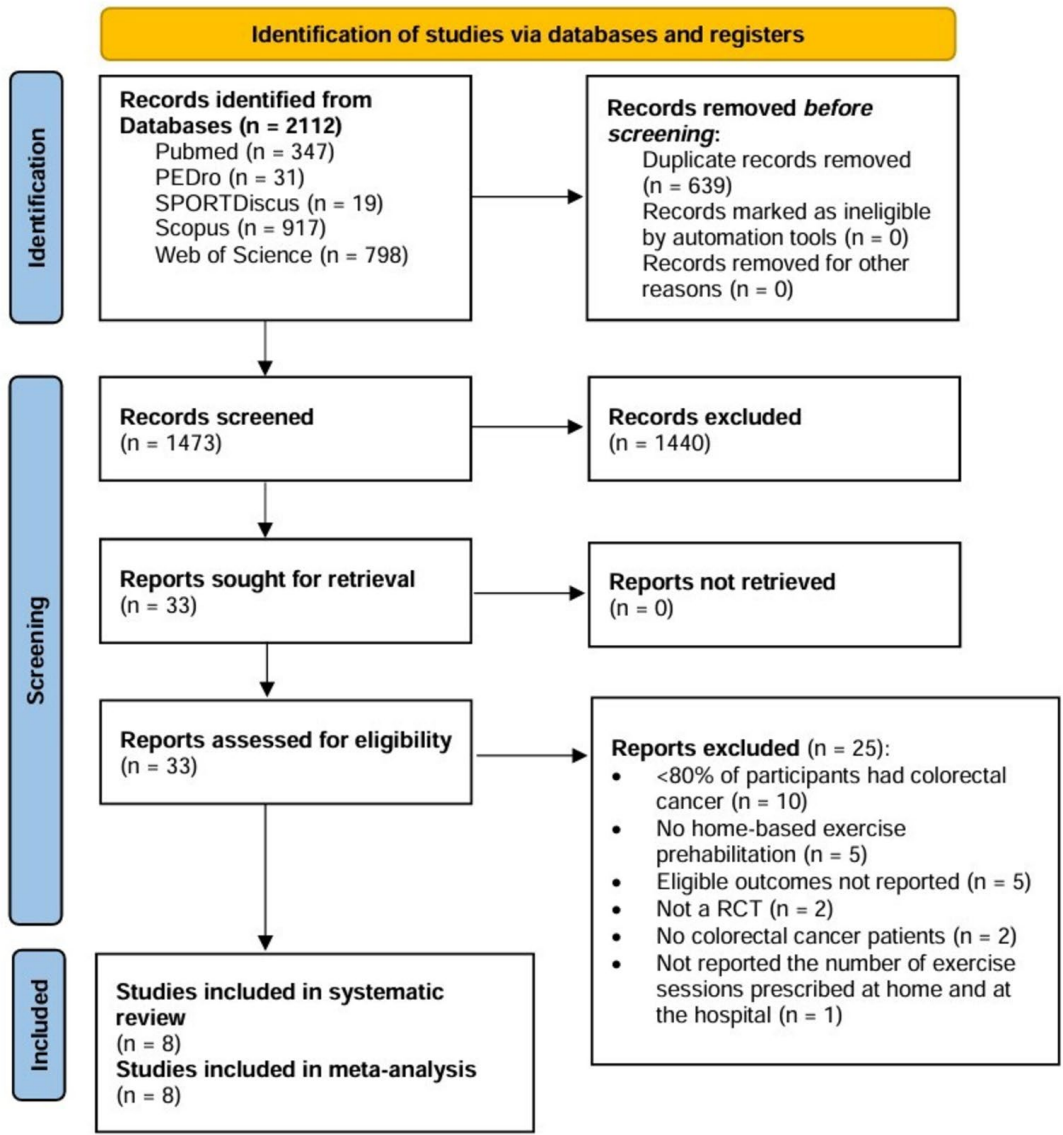
PRISMA flowchart. RCT, randomized controlled trial

The kappa statistics of the agreement between the independent reviewers was 0.73 for title/abstract screening and 0.66 for full-text screening, showing a substantial agreement. The flowchart of the literature search, screening, and selection process is presented in Fig. 1.

### Study characteristics

Table 1 summarizes the characteristics of the eligible studies. The studies included a total of 1092 participants, with 533 participants assigned to the prehabilitation group (prehab), and 559 participants assigned to the control group.

**Table 1:** Characteristics of included studies/participants

Reference	Sample size/sex/age	Tumor location	Tumor stage (prehab vs control)	Surgical approach	Prehab	Control	Relevant outcomes
Atoui et al. 2024 [5]	Prehab (*n* = 43) M = 25; F = 21 65.6 ± 12.6 years Control (*n* = 46) M = 21; F = 25 64.8 ± 14.4 years	Colon Prehab = 55.8% Control = 58.7% Rectal Prehab = 44.2% Control = 41.3%	Stage 0: 9.3% vs 6.5% Stage I–II: 46.5% vs 50% Stage III–IV: 44.2% vs 43.5%	Laparoscopic Prehab = 88.4% Control = 93.5%	Trimodal prehabilitation (exercise + nutrition + psychological intervention)	Standard of care (ERAS protocol without prehabilitation)	Exercise capacity: 6MWT Postoperative complications: Dindo–Clavien classification Length of hospital stay
Bousquet Dion et al. 2018 [11]	Prehab (*n* = 37) M = 30; F = 7 74 years [67.5–78]* Control (*n* = 26) M = 16; F = 10 71 years [54.5–74.5]*	Colon Prehab = 68% Control = 76% Rectal Prehab = 32% Control = 24%	Stage 0: 11% vs 15% Stage I–II: 59% vs 42% Stage III–IV: 30% vs 42%	Laparoscopic Prehab = 84% Control = 81%	Trimodal prehabilitation (exercise + nutrition + psychological intervention) + 8-week postoperative multimodal rehabilitation	Standard of care (ERAS protocol without prehabilitation) + 8-week postoperative multimodal rehabilitation	Exercise capacity: 6MWT Postoperative complications: Comprehensive Complication Index and Dindo–Clavien classification Length of hospital stay
Carli et al. 2020 [13]	Prehab (*n* = 55) M = 29; F = 26 78 years [72–82]* Control (*n* = 55) M = 23; F = 32 82 years [75–84]*	Colon Prehab = 67.3% Control = 76.4% Rectal Prehab = 32.7% Control = 23.6%	Stage 0–I: 32.7% vs 28.3% Stage II: 27.3% vs 34% Stage III: 34.5% vs 30.2% Stage IV: 5.5% vs 7.5%	Minimally invasive surgery** Prehab = 76.4% Control = 81.2%	Trimodal prehabilitation (exercise + nutrition + psychological intervention) + 4-week postoperative multimodal rehabilitation	Standard of care (ERAS protocol without prehabilitation) + 4-week postoperative multimodal rehabilitation	Exercise capacity: 6MWT HRQoL: SF-36 Postoperative complications: Comprehensive Complication Index and Dindo–Clavien classification Length of hospital stay
Gillis et al. 2014 [22]	Prehab (*n* = 38) M = 21; F = 17 65 ± 13.6 years Control (*n* = 39) M = 27; F = 12 66 ± 9.1 years	Prehab = 53% Control = 59% Rectal Prehab = 37% Control = 41%	Stage I–II: 55% vs 67% Stage III + : 45% vs 33%	Laparoscopic Prehab = 97% Control = 90%	Trimodal prehabilitation (exercise + nutrition + psychological intervention) + 8-week postoperative multimodal rehabilitation	Standard of care (ERAS protocol without prehabilitation) + 8-week postoperative multimodal rehabilitation	Exercise capacity: 6MWT HRQoL: SF-36 Postoperative complications: Dindo–Clavien classification Length of hospital stay
Karlsson et al. 2019 [34]	Prehab (*n* = 10) M = 4; F = 6 83.5 years [76–85]* Control (*n* = 11) M = 4; F = 6 74.0 years [73–76]*	Colon Prehab: 90% Control: 82% Rectal Prehab: 10% Control: 18%	Stage 0: 0% vs 18.2% Stage I: 0% vs 27.3% Stage II: 50% vs 9.1% Stage III: 40% vs 45.4% Stage IV: 10% vs 0%	Laparoscopic Prehab: 70% Control: 73%	Unimodal prehabilitation (aerobic + resistance exercise + Inspiratory muscle training)	Standard of care (preoperative information and advice to follow 150 min/week of moderate physical activity)	Exercise capacity: 6MWT Postoperative complications: Dindo–Clavien classification Length of hospital stay
López-Rodríguez-Arias et al. 2021 [35]	Prehab (*n* = 10) M = 4; F = 6 66.5 ± 5.6 years Control (*n* = 10) M = 3; F = 7 66 ± 8 years	Colon Prehab: 80% Control: 70% Rectal Prehab: 20% Control: 30%	T0–T1-Tis: 80% vs 50% T2–T3: 20% vs 40% T4: 0% vs 10% N0: 80% vs 70% N1: 20% vs 30%	Minimally invasive surgery Prehab = 100% Control = 100%	Trimodal prehabilitation (exercise + nutrition + relaxation exercises) + 6–8 weeks postoperative multimodal rehabilitation	Standard of care (ERAS protocol without Prehabilitation)	Postoperative complications: Dindo–Clavien classification Length of hospital stay
Onerup et al. 2022 [45]	Prehab (*n* = 317) M = 176; F = 141 68 ± 11 years Control (*n* = 351) M = 224; F = 127 69 ± 11 years	Colon Prehab:50% Control:50% Rectal Prehab:50% Control:50%	Stage I: 25% vs 25% Stage II: 23% vs 27% Stage III: 37% vs 31% Stage IV: 5% vs 7% Missing: 9% vs 9%	Laparoscopic Prehab:56% Control:52%	Unimodal prehabilitation (aerobic exercise + inspiratory muscle training) + 4 weeks postoperative aerobic exercise	Standard of care (ERAS protocol without prehabilitation)	Postoperative complications: Dindo–Clavien classification Length of hospital stay
Triguero-Cánovas et al. 2023 [58]	Prehab (*n* = 23) M = 16; F = 7 68.1 ± 7.7 years Control (*n* = 21) M = 13; F = 8 67.2 ± 8.5 years	Colon Prehab: 91.3% Control: 81% Rectal Prehab: 8.7% Control: 19%	T0–T1-Tis: 47.8% vs 19% T2–T3: 47.8% vs 57.1% T4: 4.3% vs 23.8% N0: 82.6%/61.9% N1: 17.4%/38.1%	Minimally invasive surgery Prehab = 100% Control = 100%	Trimodal prehabilitation (exercise + nutrition + relaxation exercises) + 30 days postoperative multimodal rehabilitation	Standard of care (ERAS without Prehabilitation)	Exercise capacity: 6MWT and CPET Postoperative complications: Dindo–Clavien classification Length of hospital stay

*Median age [interquartile range]

**Includes laparoscopic or transanal minimally invasive surgery (TAMIS)

*6MWT*, 6-min walk test; *ERAS*, enhanced recovery after surgery; *CPET*, cardiopulmonary exercise test; *SF-36*, 36-item short-form health survey; *F*, female; *M*, male; *prehab*, prehabilitation

The average mean/median age of participants ranged from 64.8 to 83.5 years, and the proportion of men was 58.2%. The most common tumor location was colon cancer (*n* = 627; 57%). Five studies included patients undergoing surgery via laparoscopic and open approaches [5, 11, 22, 34, 45]. One study included patients undergoing surgery via open and minimally invasive approaches (laparoscopic and transanal minimally invasive surgery) [13]. Two studies included only patients undergoing surgery via minimally invasive approaches [35, 58].

Six studies reported administration of neoadjuvant therapy (*n* = 143; 13%) [5, 11, 13, 22, 34, 45], and two studies reported administration of adjuvant therapy (*n* = 31; 3%) [11, 22].

### Intervention characteristics

A detailed description of the home-based exercise prehabilitation programs is presented in Table 2. Six studies included multimodal prehabilitation (exercise training plus nutritional and psychological interventions) [5, 11, 13, 22, 35, 58] and two studies of unimodal prehabilitation (exercise training alone) [34, 45]. The intervention period ranged from 14 to 30 days preoperatively, with most studies presenting a 4-week intervention period [5, 11, 13, 22]. In six studies the intervention continued for 4–8 weeks after surgery [11, 13, 22, 35, 45, 58].

**Table 2:** Characteristics of the home-based exercise prehabilitation programs

Reference	Modality	Time/intensity	Progression	Frequency	Program duration	Adverse events	Adherence rate
Aerobic plus resistance training							
Atoui et al. 2024 [5]	Aerobic Walking, jogging, or aerobic exercise machine Resistance 8 exercises targeting major muscle groups with elastic bands	Aerobic Duration: 20 min Intensity: RPE of 12–15 on the 20-point Borg scale Resistance Duration: 20 min Intensity: 8–12 RM	Aerobic Once the patient could complete the aerobic exercise with mild exertion (RPE of 12) Resistance The load was increased when the participant could complete 15 repetitions of a given resistance exercise	3 sessions per week	4 weeks	Not reported	85.6%
Bosquet-Dion et al. 2018 [11]	Aerobic Walking, cycling, or jogging Resistance 8 exercises targeting major muscle groups of the core, upper, and lower limbs with elastic bands	Aerobic Duration: 30 min Intensity: 60–70% HR_max_ Resistance Duration: 2 sets of 8–15 reps Intensity: not reported	Not reported	3–5 sessions per week	4 weeks	Not reported	Unclear (over 90%)
Carli et al. 2020 [13]	Aerobic Walking Resistance 8 exercises targeting major muscle groups of the core, upper, and lower limbs with elastic bands	Aerobic Duration: 30 min Intensity: 60–70% HR_max_ Resistance Duration: 2 sets of 8–15 reps Intensity: not reported	Not reported	Aerobic Daily sessions Resistance 3 sessions per week	4 weeks	No adverse events	Unclear
Gillis et al. 2014 [22]	Aerobic Walking, jogging, swimming, or cycling Resistance 8 exercises targeting major muscle groups with elastic bands	Aerobic Duration: 20 min Intensity: 40% heart rate reserve (starting intensity) Resistance Duration: 20 min Intensity: 8–12 RM	Aerobic Once the patient could complete the aerobic exercise with mild exertion (RPE of 12) Resistance The load was increased when the participant could complete 15 repetitions of a given resistance exercise	3 sessions per week (minimum)	4 weeks	Not reported	Unclear (overall adherence to the trimodal prehabilitation program: 78%)
López-Rodríguez-Arias et al. 2021 [35]	Aerobic Type of exercises not reported Resistance Type of exercises not reported	Aerobic plus resistance training Duration: 30–45 min Intensity: not reported	Not reported	Daily sessions	For 30 days before surgery	Not reported	Not reported
Triguero-Cánovas et al. 2023 [58]	Aerobic Type of exercises not reported Resistance Functional exercises adapted to the physical condition of the patient	Aerobic Duration: 30–50 min Intensity: not reported Resistance Duration: 20 min Intensity: not reported	Not reported	Aerobic: daily sessions Resistance: 3 sessions per week	Unclear (preoperative period)	Not reported	Not reported
Aerobic plus inspiratory muscle training (IMT)							
Onerup et al. 2022 [45]	Aerobic Type of exercises not reported IMT Threshold device (Philips Respironics, Eindhoven, Netherlands)	Aerobic Duration: 30 min Intensity: moderate intensity according to the Borg scale IMT Duration: 2 × 30 breaths Intensity: 30% of maximal inspiratory pressure	Not reported	Aerobic: daily sessions IMT Twice daily	For 14 days before surgery	No adverse events	Unclear (percentage of patients who reported compliance with the program ≥ 8 days: 63%)
Aerobic plus resistance and inspiratory muscle training (IMT)							
Karlsson et al. 2019 [34]	Aerobic Stair climbing, Nordic walking outdoors, and interval walking indoors and/or outdoors. Resistance Functional strength exercises such as chair stands and step-ups with weight belts. IMT Electronic device Power Breathe K3 (POWERbreathe, International Ltd., UK)	Aerobic Duration: not reported Intensity: RPE of 7–8 on Borg CR-10 Resistance Duration: 3 × 10 reps Intensity: RPE of 7–8 on Borg CR-10 IMT Duration: 30 breaths Intensity: 50% of maximum inspiratory pressure	Aerobic The duration of the session, as well as the number and length of the intervals, were increased for progression. Resistance Weight load and number of repetitions were increased if the RPE was lower than 7 on the Borg CR-10. IMT The resistance was increased by 5% if the RPE was lower than 5 on the Borg CR-10 scale.	Aerobic plus resistance training 2–3 sessions per week IMT Twice daily	For at least 2 weeks before surgery	4 adverse events: joint pain, leg pain, back pain, and dizziness.	97% (home-based supervised training sessions) Adherence to unsupervised home-based training sessions was not reported.

*Borg CR-10*, Borg category-ratio 10; *HR*_*max*_, maximal heart rate; *RM*, repetition maximum; *reps*, repetitions; *RPE*, rate of perceived exertion

The home-based exercise prehabilitation program consisted of aerobic plus resistance training in six studies (75%) [5, 11, 13, 22, 35, 58]. One study involved aerobic, resistance, and inspiratory muscle training [34], and one study involved aerobic plus inspiratory muscle training [45].

The duration of aerobic exercise was reported in six studies and varied from 20 to 50 min [5, 11, 13, 22, 45, 58]. The training intensity was reported in six studies [5, 11, 13, 22, 34, 45] and predominantly consisted of moderate-intensity aerobic exercise.

The volume of resistance training was reported in six studies and varied from 2 sets of 8–15 repetitions [11, 13] to 3 sets of 10 repetitions [34], or a duration of 20 min [5, 22, 58]. The training intensity was reported in three studies and consisted of 8–12 repetitions maximum [5, 22], or an intensity corresponding to a rate of perceived exertion of 7–8 on the Borg Category Ratio-10 scale [34].

Inspiratory muscle training was prescribed twice daily and consisted of 1–2 sets of 30 breaths at 30–50% of maximal inspiratory pressure, using a respiratory muscle trainer device [34, 45].

Two studies (25%) combined home-based and hospital-based exercise prehabilitation [11, 13]. The hospital component was supervised by a kinesiologist once a week for 4 weeks. During these sessions, patients performed 30 min of moderate-intensity aerobic exercise on a recumbent stepper or treadmill, followed by 25 min of resistance exercises targeting major muscle groups of the core, upper, and lower limbs, and concluded with 5 min of stretching.

The control groups received standard perioperative care, which predominantly consisted of treatment along an Enhanced Recovery After Surgery (ERAS) pathway without prehabilitation.

### Methodological quality assessment

Overall, the quality assessment showed a mean PEDro score of 6.5 ± 1.4, indicating a good methodological quality. A total of six studies showed good methodological quality (PEDro score: 6 to 8) [5, 11, 13, 22, 34, 45] and two studies showed a reasonable methodological quality (PEDro score: 4 and 5) [35, 58].

Due to the nature of the intervention, blinding of participants and therapists was not performed in any of the included studies. In six studies (75%), intention-to-treat analysis was not performed [5, 11, 22, 34, 35, 58]. In two studies (25%) hidden allocation was not performed and outcome assessors were not blinded to treatment assignment [35, 58]. In two studies (25%), there was no adequate follow-up [11, 58]. The methodological quality of the included studies is presented in Table 3.

**Table 3:** Methodological quality assessment using the PEDro scale

Reference	Eligibility criteria*	Randomized allocation	Hidden allocation	Baseline comparison between groups	Blind participants	Blind physical therapists	Blind assessors	Proper follow-up	Intention to treat analysis	Comparison between groups	Point estimate and variability	Total score
Atoui et al. 2024 [5]	✓	✓	✓	✓	X	X	✓	✓	X	✓	✓	7/10
Bousquet-Dion et al. 2018 [11]**	✓	✓	✓	✓	X	X	✓	X	X	✓	✓	6/10
Carli et al. 2020 [13]	✓	✓	✓	✓	X	X	✓	✓	✓	✓	✓	8/10
Gillis et al. 2014 [22]	✓	✓	✓	✓	X	X	✓	✓	X	✓	✓	7/10
Karlsson et al. 2019 [34]	✓	✓	✓	✓	X	X	✓	✓	X	✓	✓	7/10
López-Rodríguez-Arias et al. 2021 [35]	✓	✓	X	✓	X	X	X	✓	X	✓	✓	5/10
Onerup et al. 2022 [45]	✓	✓	✓	✓	X	X	✓	✓	✓	✓	✓	8/10
Triguero Canovas et al. 2023 [58]	✓	✓	X	✓	X	X	X	X	X	✓	✓	4/10

*Eligibility criteria item does not contribute to the total score

**Intention to treat analysis was presented for length of hospital stay but not for 6-min walk distance (primary outcome) and postoperative complications

### Synthesis of the results

A total of 8 studies (*n* = 1092 participants) [5, 11, 13, 22, 34, 35, 45, 58] were pooled to assess the effects of home-based exercise prehabilitation on postoperative outcomes after colorectal cancer surgery.

#### Primary outcome: effect of home-based exercise prehabilitation on exercise capacity

**Fig. 2:**
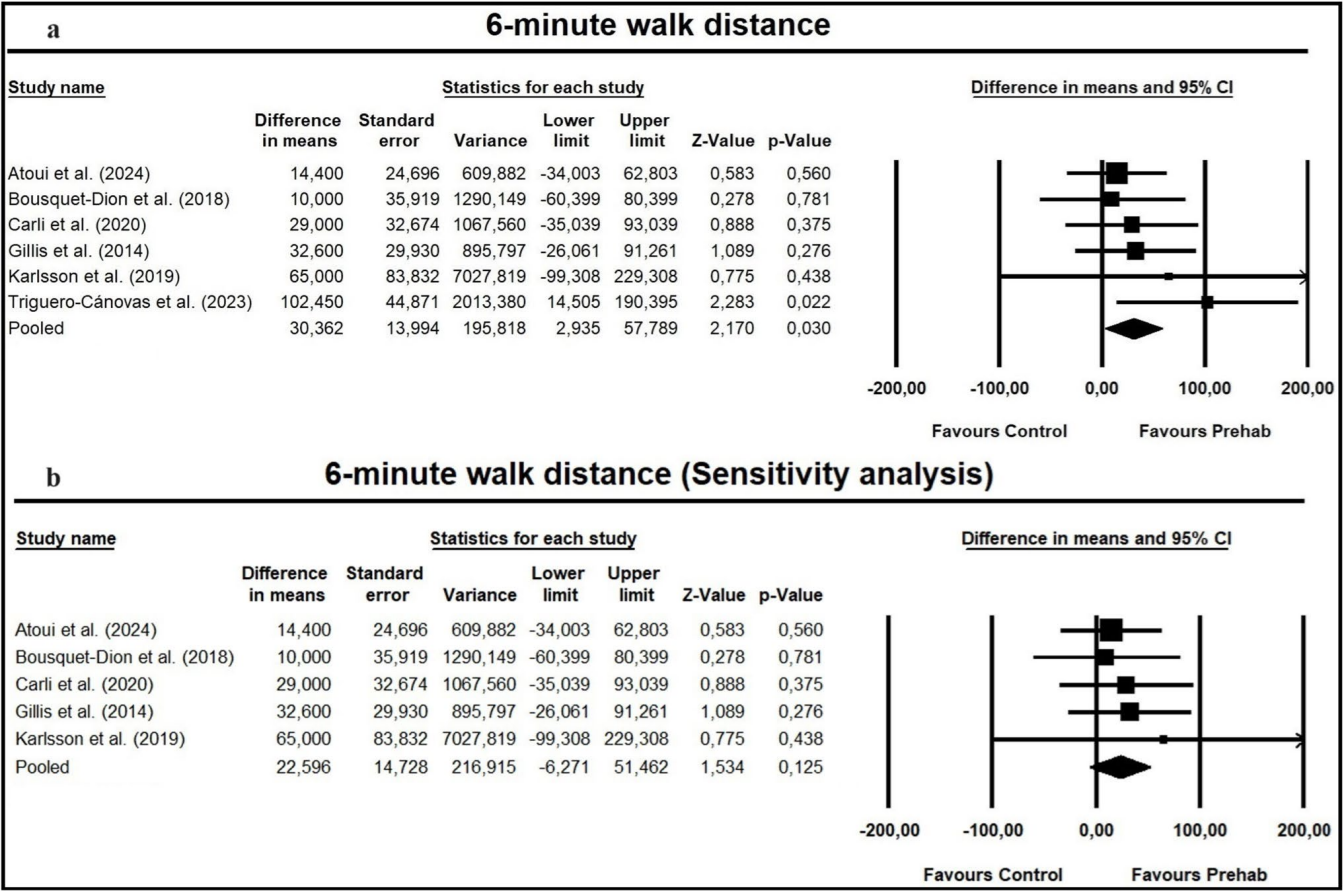
**a** Meta-analysis for the effect estimate on 6-min walk distance. **b** Sensitivity analysis for the effect estimate on 6-min walk distance, including only studies with a PEDro score ≥ 6. *CI*, confidence interval

Six studies [5, 11, 13, 22, 34, 58] reported functional exercise capacity, measured by the 6MWT. Postoperative assessments were conducted at hospital discharge in one study [34], 4 weeks after surgery in four studies [5, 11, 13, 22], and 6 weeks after surgery in one study [58]. The meta-analysis showed that home-based exercise prehabilitation results in a significant improvement in postoperative 6-min walk distance compared with controls (MD = 30.36: 95% CI: [2.94; 57.59]; *Z* = 2.17; *p* = 0.03; *I*^2^ = 0%) (Fig. 2a). However, sensitivity analysis including only studies with good methodological quality (PEDro score ≥ 6) revealed no significant effects (MD = 22.60: 95% CI [-6.27; 51.46]; Z = 1.53; *p* = 0.13; I^2^ = 0%) (Fig. 2b).

#### Secondary outcomes: effect of home-based exercise prehabilitation on postoperative complications

**Fig. 3:**
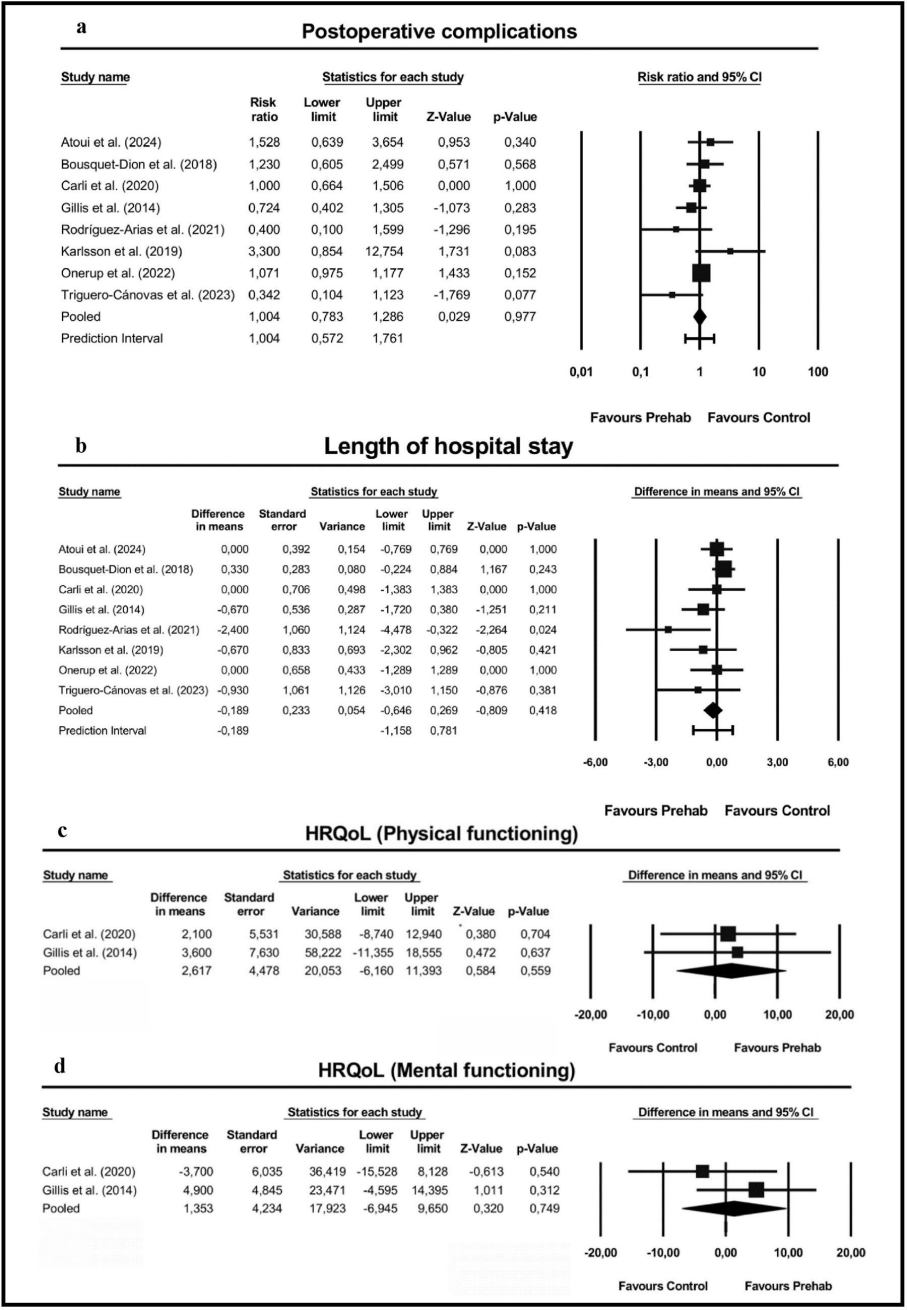
**a** Meta-analysis for the effect estimate on postoperative complications. **b** Meta-analysis for the effect estimate on length of hospital stay. **c** Meta-analysis for the effect estimate on physical functioning. **d** Meta-analysis for the effect estimate on mental functioning. *CI*, confidence interval

Eight studies [5, 11, 13, 22, 34, 35, 45, 58] reported the number of participants who developed postoperative complications. Data were collected from medical records within the first 30 days after surgery, according to the Clavien–Dindo classification. The meta-analysis showed no significant effect of home-based exercise prehabilitation on postoperative complications (RR = 1.00: 95% CI: [0.78; 1.29]; *Z* = 0.03; *p* = 0.98; *I*^2^ = 34.35) (Fig. 3a). Similar results were found in sensitivity analysis of studies with good methodological quality (RR = 1.07: 95% CI: [0.94; 1.21]; *Z* = 1.04; *p* = 0.30;* I*^2^ = 4.72%) (Supplementary Fig. 1a).

#### Effect of home-based exercise prehabilitation on length of hospital stay

Eight studies [5, 11, 13, 22, 34, 35, 45, 58] reported postoperative length of hospital stay. The meta-analysis showed no significant effect of home-based exercise prehabilitation on length of hospital stay (MD = − 0.20: 95% CI: [− 0.65; 0.23]; *Z* = − 0.81; *p* = 0.42; *I*^2^ = 24.86%) (Fig. 3b). Similar results were found in sensitivity analysis of studies with good methodological quality (MD = 0.03: 95% CI: [− 0.34; 0.40]; *Z* = 0.16; *p* = 0.88;* I*^2^ = 0%) (Supplementary Fig. 1b).

#### Effect of home-based exercise prehabilitation on HRQoL

Two studies [13, 22] reported HRQoL, measured by the 36-item short form health survey (SF-36), at 4 weeks after surgery. The meta-analysis showed no significant effect of home-based exercise prehabilitation on physical functioning (MD = 2.62: 95% CI: [− 6.16; 11.39]; *Z* = 0.58; *p* = 0.56; *I*^2^ = 0%) and mental functioning (MD = 1.35: 95% CI: [− 6.95; 9.65]; Z = 0.32; *p* = 0.75; *I*^2^ = 19.02%) (Fig. 3c and 3d).

### Publication bias

The funnel plot was asymmetrical for 6-min walk distance and length of hospital stay, suggesting the possibility of publication bias (Supplementary Fig. 2). For 6-min walk distance, the Egger’s test showed an intercept result of 1.48 (SE = 1.00; 95% CI: [− 1.29; 4.25]; *t* = 1.48; *p* = 0.21), indicating no evidence of publication bias (Supplementary Fig. 3a). For length of hospital stay, the Egger’s test showed an intercept result of − 2.03 (SE = 0.59; 95% CI: [− 3.48; − 0.58]; *t* = 3.42; *p* = 0.01), confirming a strong evidence of publication bias (Supplementary Fig. 3c). No evidence of publication bias was found for postoperative complications (Supplementary Fig. 2). For HRQoL, due to the limited number of included studies, we were not able to generate funnel plots.

### GRADE assessment

The GRADE assessment is summarized in Table 4.

**Table 4:** GRADE assessment

Participants (studies)	Risk of bias	Inconsistency	Indirectness	Imprecision	Publication bias	Overall certainty of evidence
Exercise capacity						
428 (6 RCTs)	Serious	Not serious	Not serious	Serious	None	⨁⨁◯◯ Low
Postoperative complications						
1092 (8 RCTs)	Not serious	Serious	Not serious	Not serious	None	⨁⨁⨁◯ Moderate
Length of hospital stay						
1092 (8 RCTs)	Not serious	Serious	Not serious	Not serious	None	⨁⨁⨁◯ Moderate
HRQoL (physical functioning)						
145 (2 RCTs)	Serious	Not serious	Not serious	Serious	None	⨁⨁◯◯ Low
HRQoL (Mental functioning)						
145 (2 RCTs)	Serious	Serious	Not serious	Serious	None	⨁◯◯◯ Very low

#### Risk of bias

For exercise capacity, postoperative complications, and length of hospital stay, the absence of allocation concealment and blinded outcome assessors in two studies [35, 58] may have influenced the treatment effects. After excluding these studies from the sensitivity analysis, the overall effect estimate changed significantly for the 6-min walk distance (Fig. 2b) but not for postoperative complications and length of hospital stay (Supplementary Fig. 1). Therefore, we decided to downgrade certainty of evidence for risk of bias exclusively for the 6-min walk distance.

For HRQoL, we decided to downgrade certainty of evidence for risk of bias due to unblinded participants in the two studies included in the meta-analysis [13, 22].

#### Imprecision

For exercise capacity, the result was downgraded for imprecision because the 95% CI (2.94 to 57.59) crosses the threshold of 19 m considered clinically meaningful in patients undergoing abdominal surgery [3] (Table 4).

For postoperative complications and length of hospital stay, imprecision was considered not important because a relatively narrow 95% CI was found for the overall effect estimate.

For HRQoL, the estimated was downgraded for imprecision due to small sample sizes and a wide 95% CI both to physical functioning and mental functioning.

#### Inconsistency

For exercise capacity, inconsistency was considered not important because heterogeneity was low (*I*^2^ = 0%), all studies showed the same direction of the effect, and differences in the point estimate between studies with good methodological quality were small.

For length of hospital stay and postoperative complications, we decided to downgrade certainty of evidence because, even though statistical heterogeneity was small (*I*^2^ < 35%), the variability in the magnitude and direction of the effect estimates between studies suggests inconsistency.

For HRQoL, inconsistency was downgraded for mental functioning as result of variability in the effect estimates between studies. For physical functioning, inconsistency was considered not important.

#### Indirectness

Indirectness was considered not important because the effect estimate resulted from direct evidence of RCTs, where the population, intervention, and outcomes did not differ from those of interest.

#### Publication bias

For exercise capacity and postoperative complications, publication bias was considered not important.

For length of hospital stay, significant evidence of publication bias was found by the Egger’s test (Supplementary Fig. 3). However, sensitivity analysis of studies with good methodological quality showed no evidence of publication bias (Supplementary Fig. 4). Since the overall effect estimate was not statistically significant in both analyses, indicating that findings were unlikely to be influenced by publication bias, we decided not to downgrade the certainty of evidence.

For HRQoL, evaluating publication bias would be speculative due to the insufficient number of studies to create a funnel plot. Therefore, we decided not to downgrade the evidence.

## Discussion

This systematic review and meta-analysis examined the effects of home-based exercise prehabilitation on postoperative outcomes in colorectal cancer patients. Findings suggest that home-based exercise prehabilitation does not reduce postoperative complications and length of hospital stay after colorectal cancer surgery. Its effect on postoperative exercise capacity and HRQoL is uncertain due to low-quality evidence.

In terms of postoperative exercise capacity, the primary analysis revealed beneficial effects of home-based exercise prehabilitation. However, after excluding a study with high overall risk of bias [58], the sensitivity analysis showed no significant effects (MD = 22.60: 95% CI: [− 6.27; 51.46]). This discrepancy suggests that the positive effects observed in the primary analysis might have been influenced by some methodological limitations presented in this study, such as the lack of concealed allocation and blinded outcome assessors, which may overestimate treatment effects [4, 49]. Furthermore, the prehabilitation group in this study received postoperative multimodal rehabilitation [58], which was not provided to the control group, potentially influencing the results. Therefore, while the initial findings were promising, the sensitivity analysis indicated that these results should be interpreted with caution.

For postoperative complications and length of hospital stay, moderate evidence from eight studies showed no benefit of home-based exercise prehabilitation. These findings did not change in the sensitivity analysis including studies with good methodological quality. Moreover, although we did not investigate the effects on specific types of complications, no significant between-group differences were found for the different types of complications in the included studies. Collectively, these results suggest that home-based exercise prehabilitation is ineffective in improving these surgical outcomes after colorectal cancer surgery.

A previous systematic review concluded that exercise-based prehabilitation has no effects on length of hospital stay, but significantly improves exercise capacity and might reduce postoperative complications in colorectal cancer patients [19]. However, the comparison between our results and this study is limited because it focused on exercise capacity prior to surgery and included patients with benign colorectal lesions and other abdominal neoplasms. More importantly, the effect estimates in this systematic review combined home-based unsupervised exercise interventions and supervised moderate-to-high intensity interval training programs delivered at the hospital and in community-based settings. This heterogeneity in the exercise interventions hindered a clear understanding of which exercise regimens are effective for patients undergoing colorectal cancer surgery.

It is possible that, within the limited time available for prehabilitation, low-to-moderate intensity home-based exercise training may not provide a sufficient stimulus to induce clinically meaningful adaptations [57, 65], which could explain the lack of effects observed in our meta-analysis. Therefore, a higher training intensity may be required to improve functional recovery and surgical outcomes. This hypothesis is supported by previous clinical trials involving rectal cancer patients, which demonstrated significant improvements in cardiorespiratory fitness following 6–9 weeks of preoperative in-hospital high-intensity interval training (HIIT) [36, 66]. Additionally, Berkel et al. (2022) found that 3 weeks of a community-based prehabilitation program involving moderate-to-high intensity interval training plus resistance training significantly improved exercise capacity and reduced postoperative complications in high-risk patients scheduled for elective colon resection [7]. In a prehabilitation trial comparing the effect of in-hospital HIIT with moderate intensity continuous training (MICT) in colorectal cancer patients, while both programs significantly enhanced preoperative exercise capacity, peak oxygen uptake in the HIIT group was 1.51 mL kg^−1^ min^−1^ higher than in the MICT group (95% CI: [− 0.19; 3.20]) [40]. More importantly, at 2 months after surgery, the HIIT group exhibited a greater improvement in oxygen uptake at the anaerobic threshold compared to the MICT group, with a between-group difference of 2.36 mL kg^−1^ min^−1^ (95% CI: [0.378; 4.34]) [40]. Similarly, 4 weeks of HIIT was found to elicit significant improvements in peak oxygen uptake among colorectal cancer survivors, whereas no significant changes were observed after 4 weeks of MICT [16, 17].

The above findings suggest that within a short time frame, a higher training intensity may be needed to enhance patients' physiological reserve, potentially improving their resilience to cope with the surgical stress. However, further research is warranted to establish the optimal dose for exercise-based prehabilitation programs to improve postoperative outcomes in colorectal cancer patients. This requires a detailed description of the training programs, which is a limitation of many studies included in our meta-analysis, as key components such as exercise intensity [35, 58] and progression [11, 13, 35, 45, 58] were not specified.

Another factor that should be considered when interpreting intervention effects is the absence or incomplete reporting of exercise adherence across studies. This limitation is significant, as adherence rates directly influence training effects and remain a challenge in exercise-based prehabilitation trials [20]. Therefore, a close examination of adherence rates is essential in future studies, along with a transparent description of current reporting standards in exercise oncology [18, 43]. Moreover, given that adherence is often poor in home-based prehabilitation trials [14, 20], the use of digital health technologies to deliver these programs may provide a solution that can enhance patients’ self-efficacy, leading to higher exercise adherence [50]. This approach has been shown to be feasible, well accepted and effective in patients undergoing major surgery [8, 47]. However, its potential in oncological colorectal surgery remains unknown and warrants future research.

Despite strong evidence suggesting a positive impact of exercise training on the HRQoL of cancer patients [12, 21], the effect of home-based exercise prehabilitation on HRQoL after colorectal cancer surgery remains uncertain due to the limited number of studies and small sample sizes. Thus, further adequately powered clinical trials are needed to elucidate the effects of home-based exercise prehabilitation on HRQoL. These trials should use cancer-specific questionnaires, such as the EORTC-QLQ-CR29 [68], to better capture the unique symptomatic and functional concerns experienced by colorectal cancer patients [68].

The strengths of this systematic review and meta-analysis include rigorous adherence to PRISMA guidelines [41] and the extensive literature search across multiple databases. Furthermore, unlike previous systematic reviews in this field [9, 19], we exclusively included RCTs, used the GRADE approach to assess the certainty of evidence, and excluded studies enrolling more than 20% of patients with benign disease or other abdominal neoplasms, thus mitigating clinical heterogeneity.

However, some limitations need to be acknowledged. Firstly, despite multiple attempts to contact the authors of the studies, we were unable to obtain the mean and standard deviation for length of hospital stay in five studies [5, 11, 13, 22, 34]. Hence, although we used validated methods to estimate these values [32, 61], this limitation may have restricted a more precise estimation of the exercise effect. Secondly, our eligibility criteria focused on aerobic and resistance training. This decision was based on strong evidence supporting their benefits in cancer patients [12, 37], and aimed to reduce heterogeneity in the type of exercise interventions; however, it limited our ability to evaluate the effects of other training modalities, such as pelvic floor muscle training and respiratory muscle training. Thirdly, the generalization of our findings to patients who may have a greater need for preoperative optimization is limited because only two studies focused on these individuals (older adults [34] and frail patients [13]), which precluded a subgroup analysis. Lastly, we confined our search to studies published in English, Spanish and Portuguese, which may have influenced our findings.

## Conclusion

This systematic review and meta-analysis concluded that home-based exercise prehabilitation does not confer a significant reduction in postoperative complications and length of hospital stay after colorectal cancer surgery. Additionally, the effects on postoperative exercise capacity and HRQoL are uncertain due to low-quality evidence.

These findings indicate that there is still insufficient evidence to support home-based exercise training as a prehabilitation component in oncological colorectal surgery.

## Supplementary Information

Below is the link to the electronic supplementary material.

Supplementary file1 (PDF 501 KB)

## Data Availability

The data supporting the conclusions of this article are included in the manuscript and in its supplementary files.
