# Supplementary material for: Effect of home-based exercise prehabilitation on postoperative outcomes in colorectal cancer surgery: a systematic review and meta-analysis
Source: Support Care Cancer. 2024 Dec 12;33(1):20. doi: 10.1007/s00520-024-09069-y (PMC11635004; doi:10.1007/s00520-024-09069-y)
Supplement: Supplementary file 1 — Supplementary file1 (PDF 501 KB) [file 520_2024_9069_MOESM1_ESM.pdf]

Effect of home-based exercise prehabilitation on postoperative outcomes in colorectal cancer surgery: A systematic review and meta-analysis

Pedro Machado[1,2,3], André Paixão[4], Bárbara Oliveiros[5,6,7], Raul A. Martins[2], and Joana Cruz[1]

Institutional affiliations

[1]Center for Innovative Care and Health Technology (ciTechCare), School of Health Sciences of the Polytechnic of Leiria (ESSLei), Leiria, Portugal

[2]University of Coimbra, Research Unit for Sport and Physical Activity (CIDAF, UID/PTD/04213/2019), Faculty of Sport Sciences and Physical Education, Coimbra, Portugal

[3]Physioclem, Physical therapy clinics, Alcobaça, Portugal

[4]Sport Sciences School of Rio Maior (ESDRM), Santarém Polytechnic University

[5]University of Coimbra, Laboratory of Biostatistics and Medical Informatics (LBIM), Faculty of Medicine, Coimbra, Portugal

[6]University of Coimbra, Coimbra Institute for Clinical and Biomedical Research (iCBR), Faculty of Medicine, Portugal

[7]University of Coimbra, Institute for Biomedical Imaging and Translational Research (CIBIT), Coimbra, Portugal

Corresponding author information:

Pedro Filipe André Machado

ciTechCare – Center for Innovative Care and Health Technology; Rua de Santo André – 66-68, Campus 5, Politécnico de Leiria, 2410-541 Leiria, Portugal; pedro.machado@ipleiria.pt

ORCID: 0000-0003-1147-2778

Supplementary table 1. Database search strategy

Electronic search details in PubMed database:<br><br>(((("Colorectal Neoplasms"[MeSH Major Topic] OR ("Rectal Neoplasm"[Title/Abstract] OR "Rectal Cancer"[Title/Abstract] OR "Colon Neoplasm"[Title/Abstract] OR "Colon Cancer"[Title/Abstract] OR "abdominal"[Title/Abstract] OR "bowel"[Title/Abstract] OR "colorectal"[Title/Abstract])) AND (Surgery OR Surgical OR Resection OR Operable)) AND ("exercise"[Mesh] OR "resistance"[Title/Abstract] OR "aerobic"[Title/Abstract] OR "strength training"[Title/Abstract] OR "endurance training"[Title/Abstract] OR "interval training"[Title/Abstract] OR "physical training"[Title/Abstract] OR "high-intensity training"[Title/Abstract] OR "walking"[Title/Abstract] OR "prehabilitation"[Title/Abstract] OR (preoperative exercise[MeSH Major Topic])) AND (randomized[Title/Abstract] OR "randomised"[Title/Abstract]))) AND (clinicaltrial[Filter])
Eletronic search details in Web of Knowledge:<br>1. (“Colorectal Tumor” OR “Colorectal Neoplasm” OR “Colorectal Cancer” OR “Colorectal Carcinoma” OR “Rectal Neoplasm” OR “Rectal Cancer” OR “Colon Neoplasm” OR “Colon Cancer” OR “abdominal” OR “bowel” OR “colorectal”): TOPIC<br>2. (Surgery OR Surgical OR Resection OR operable): TOPIC<br>3. (exercise OR prehabilitation OR resistance OR “strength training” OR aerobic OR “endurance training” OR “interval training” OR “physical training” OR “high-intensity training” OR walking): TOPIC<br>4. (randomized OR randomised): TOPIC<br>Combination: 1 AND 2 AND 3 AND 4<br>Limited to: Article, English and Spanish Language
Electronic search details in PEDro database:<br>Filters applied: Subdiscipline (Oncology); Method (Clinical trial)<br>Search 1<br>Abstract and title: colorectal surgery exercise*<br>Search 2<br>Abstract and title: colorectal surgery prehabilitation<br>Search 3<br>Abstract and title: colon surgery exercise*<br>Search 4<br>Abstract and title: colon surgery prehabilitation<br>Search 5<br>Abstract and title: rectal surgery exercise*<br>Search 6<br>Abstract and title: rectal surgery prehabilitation

Supplementary Table 1. *Cont.*

Eletronic search details in Scopus:<br>1. (“Colorectal Tumor” OR “Colorectal Neoplasm” OR “Colorectal Cancer” OR “Colorectal Carcinoma” OR “Rectal Neoplasm” OR “Rectal Cancer” OR “Colon Neoplasm” OR “Colon Cancer” OR “abdominal” OR “bowel” OR “colorectal”): TITLE-ABS-KEY<br>2. (Surgery OR Surgical OR Resection OR operable): TITLE-ABS-KEY<br>3. (exercise OR prehabilitation OR resistance OR “strength training” OR aerobic OR “endurance training” OR “interval training” OR “physical training” OR “high-intensity training” OR walking): TITLE-ABS-KEY<br>4. (randomized OR randomised): TITLE-ABS-KEY<br>Combination: 1 AND 2 AND 3 AND 4<br>Limited to: Article; Human, Humans; English, Portuguese and Spanish language
Eletronic search details in SPORTdiscus<br>1. (“Colorectal Tumor” OR “Colorectal Neoplasm” OR “Colorectal Cancer” OR “Colorectal Carcinoma” OR “Rectal Neoplasm” OR “Rectal Cancer” OR “Colon Neoplasm” OR “Colon Cancer” OR abdominal OR bowel OR colorectal) Abstract or Author-supplied abstract<br>2. (Surgery OR Surgical OR Resection OR “operable”) Abstract or Author-supplied abstract<br>3. (exercise OR prehabilitation OR resistance OR “strength training” OR aerobic OR “endurance training” OR “interval training” OR “physical training” OR “high-intensity training” OR walking) AB Abstract or Author-supplied abstract<br>4. (“randomized” OR randomized) TX All Text<br>Combination: 1 AND 2 AND 3 AND 4

a

Postoperative complications (Sensitivity analysis)

Study name	Risk ratio	Lower limit	Upper limit	Z-Value	p-Value
Atoui et al. (2024)	1,528	0,639	3,654	0,953	0,340
Bousquet-Dion et al. (2018)	1,230	0,605	2,499	0,571	0,568
Carli et al. (2020)	1,000	0,664	1,506	0,000	1,000
Gillis et al. (2014)	0,724	0,402	1,305	-1,073	0,283
Karlsson et al. (2019)	3,300	0,854	12,754	1,731	0,083
Onerup et al. (2022)	1,071	0,975	1,177	1,433	0,152
Pooled	1,069	0,943	1,211	1,044	0,296
Prediction Interval	1,069	0,850	1,343		

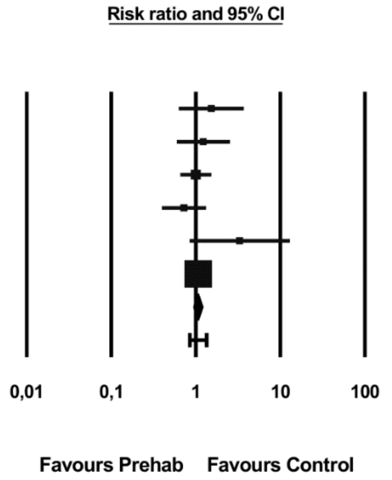

b

Length of hospital stay (Sensitivity analysis)

Study name	Difference in means	Standard error	Variance	Lower limit	Upper limit	Z-Value	p-Value
Atoui et al. (2024)	0,000	0,392	0,154	-0,769	0,769	0,000	1,000
Bousquet-Dion et al. (2018)	0,330	0,283	0,080	-0,224	0,884	1,167	0,243
Carli et al. (2020)	0,000	0,706	0,498	-1,383	1,383	0,000	1,000
Gillis et al. (2014)	-0,670	0,536	0,287	-1,720	0,380	-1,251	0,211
Karlsson et al. (2019)	-0,670	0,833	0,693	-2,302	0,962	-0,805	0,421
Onerup et al. (2022)	0,000	0,658	0,433	-1,289	1,289	0,000	1,000
Pooled	0,029	0,188	0,035	-0,340	0,398	0,155	0,877
Prediction Interval							

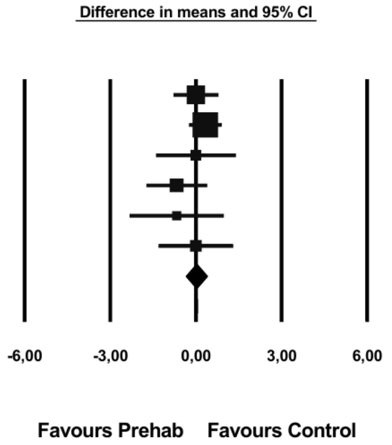

Supplementary Fig. 1 Sensitive analysis: (a) postoperative complications; (b) Length of hospital stay

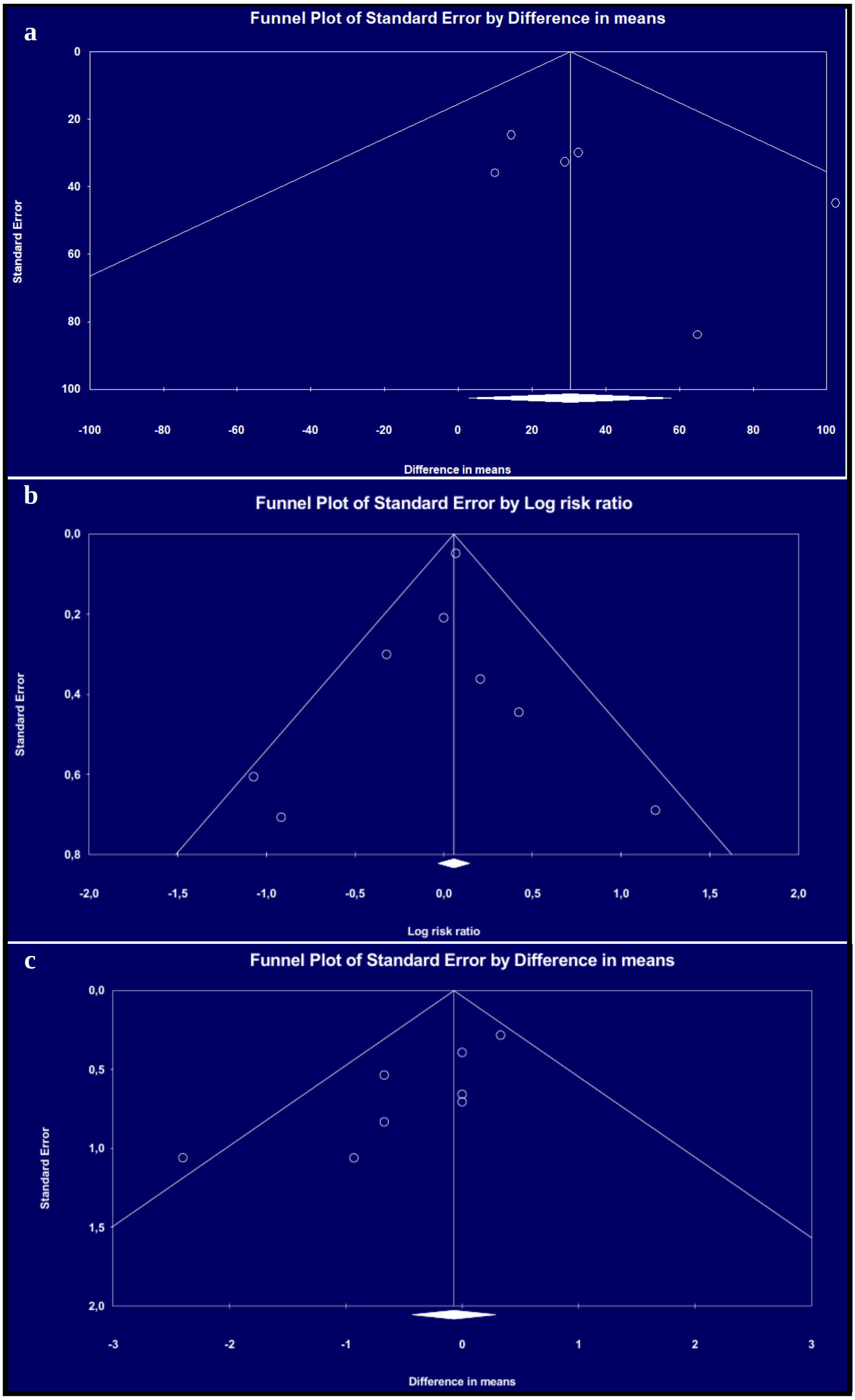

Supplementary Fig. 2 Funnel Plot: (a) 6-minute walk distance; (b) postoperative complications; (c) Length of hospital stay

a **Egger's regression intercept**

Intercept	1,48022
Standard error	0,99706
95% lower limit (2-tailed)	-1,28806
95% upper limit (2-tailed)	4,24850
t-value	1,48459
df	4,00000
P-value (1-tailed)	0,10591
P-value (2-tailed)	0,21182

b **Egger's regression intercept**

Intercept	-0,33005
Standard error	0,58281
95% lower limit (2-tailed)	-1,75613
95% upper limit (2-tailed)	1,09603
t-value	0,56631
df	6,00000
P-value (1-tailed)	0,29586
P-value (2-tailed)	0,59172

c **Egger's regression intercept**

Intercept	-2,02996
Standard error	0,59313
95% lower limit (2-tailed)	-3,48129
95% upper limit (2-tailed)	-0,57863
t-value	3,42246
df	6,00000
P-value (1-tailed)	0,00705
P-value (2-tailed)	0,01410

Supplementary Fig 3. Egger’s test: (a) 6-minute walk distance; (b) postoperative complications; (c) Length of hospital stay

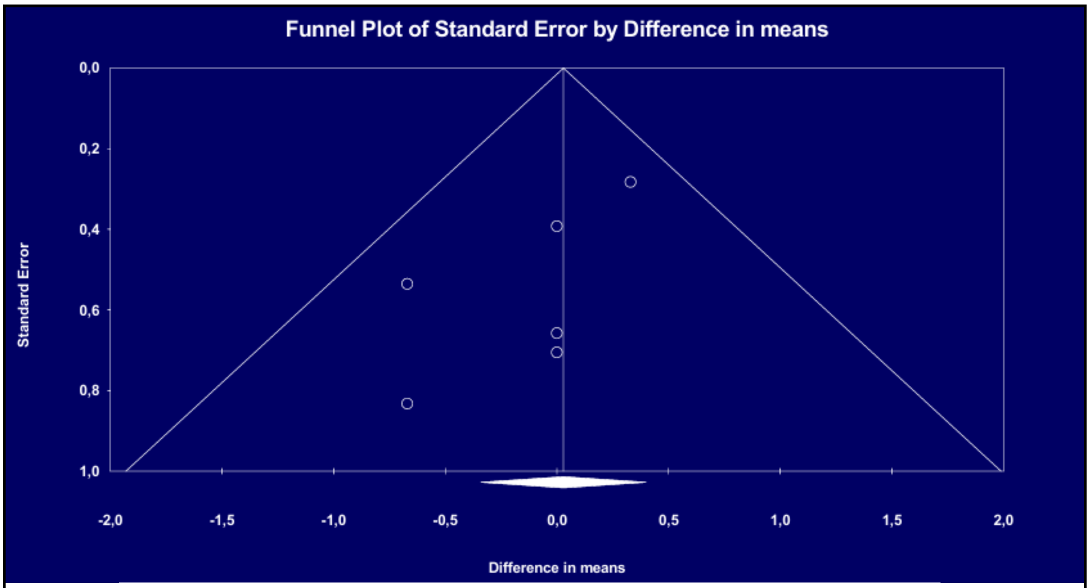

Egger's regression intercept

Intercept	-1,50453
Standard error	0,71391
95% lower limit (2-tailed)	-3,48666
95% upper limit (2-tailed)	0,47760
t-value	2,10746
df	4,00000
P-value (1-tailed)	0,05139
P-value (2-tailed)	0,10278

Supplementary Fig. 4. Funnel plot for sensitivity analysis of length of hospital stay

Supplementary Table 2 PRISMA Checklist

Section and Topic	Item #	Checklist item	Location where item is reported
**TITLE**			
Title	1	Identify the report as a systematic review.	Page 1
**ABSTRACT**			
Abstract	2	See the PRISMA 2020 for Abstracts checklist.	Page 2
**INTRODUCTION**			
Rationale	3	Describe the rationale for the review in the context of existing knowledge.	Pages 3-4
Objectives	4	Provide an explicit statement of the objective(s) or question(s) the review addresses.	Page 4
**METHODS**			
Eligibility criteria	5	Specify the inclusion and exclusion criteria for the review and how studies were grouped for the syntheses.	Pages 4-5
Information sources	6	Specify all databases, registers, websites, organisations, reference lists and other sources searched or consulted to identify studies. Specify the date when each source was last searched or consulted.	Page 5
Search strategy	7	Present the full search strategies for all databases, registers and websites, including any filters and limits used.	Page 6 and Supplementary Table 1
Selection process	8	Specify the methods used to decide whether a study met the inclusion criteria of the review, including how many reviewers screened each record and each report retrieved, whether they worked independently, and if applicable, details of automation tools used in the process.	Page 6
Data collection process	9	Specify the methods used to collect data from reports, including how many reviewers collected data from each report, whether they worked independently, any processes for obtaining or confirming data from study investigators, and if applicable, details of automation tools used in the process.	Page 6
Data items	10a	List and define all outcomes for which data were sought. Specify whether all results that were compatible with each outcome domain in each study were sought (e.g. for all measures, time points, analyses), and if not, the methods used to decide which results to collect.	Page 5
	10b	List and define all other variables for which data were sought (e.g. participant and intervention characteristics, funding sources). Describe any assumptions made about any missing or unclear information.	Pages 5-6
Study risk of bias assessment	11	Specify the methods used to assess risk of bias in the included studies, including details of the tool(s) used, how many reviewers assessed each study and whether they worked independently, and if applicable, details of automation tools used in the process.	Page 6
Effect measures	12	Specify for each outcome the effect measure(s) (e.g. risk ratio, mean difference) used in the synthesis or presentation of results.	Page 7

Supplementary Table 2 *Cont.*

Section and Topic	Item #	Checklist item	Location where item is reported
**METHODS**			
Synthesis methods	13a	Describe the processes used to decide which studies were eligible for each synthesis (e.g. tabulating the study intervention characteristics and comparing against the planned groups for each synthesis (item #5)).	-
	13b	Describe any methods required to prepare the data for presentation or synthesis, such as handling of missing summary statistics, or data conversions.	Page 7
	13c	Describe any methods used to tabulate or visually display results of individual studies and syntheses.	Page 7
	13d	Describe any methods used to synthesize results and provide a rationale for the choice(s). If meta-analysis was performed, describe the model(s), method(s) to identify the presence and extent of statistical heterogeneity, and software package(s) used.	Page 7
	13e	Describe any methods used to explore possible causes of heterogeneity among study results (e.g. subgroup analysis, meta-regression).	Page 7
	13f	Describe any sensitivity analyses conducted to assess robustness of the synthesized results.	Page 7
Reporting bias assessment	14	Describe any methods used to assess risk of bias due to missing results in a synthesis (arising from reporting biases).	Page 7
Certainty assessment	15	Describe any methods used to assess certainty (or confidence) in the body of evidence for an outcome.	Page 8
**RESULTS**			
Study selection	16a	Describe the results of the search and selection process, from the number of records identified in the search to the number of studies included in the review, ideally using a flow diagram.	Page 8 and Figure 1
	16b	Cite studies that might appear to meet the inclusion criteria, but which were excluded, and explain why they were excluded.	Figure 1
Study characteristics	17	Cite each included study and present its characteristics.	Page 9, Table 1 and Table 2
Risk of bias in studies	18	Present assessments of risk of bias for each included study.	Page 10 and Table 3
Results of individual studies	19	For all outcomes, present, for each study: (a) summary statistics for each group (where appropriate) and (b) an effect estimate and its precision (e.g. confidence/credible interval), ideally using structured tables or plots.	Figure 2, Figure 3, Supplementary Figure 1

Supplementary Table 2 *Cont.*

Section and Topic	Item #	Checklist item	Location where item is reported
Results of syntheses	20a	For each synthesis, briefly summarise the characteristics and risk of bias among contributing studies.	Pages 20-21
	20b	Present results of all statistical syntheses conducted. If meta-analysis was done, present for each the summary estimate and its precision (e.g. confidence/credible interval) and measures of statistical heterogeneity. If comparing groups, describe the direction of the effect.	Pages 17-18, Figure 2, Figure 3, Supplementary Figure 1
	20c	Present results of all investigations of possible causes of heterogeneity among study results.	Pages 17-18, Figure 2b, Supplementary Figure 1
	20d	Present results of all sensitivity analyses conducted to assess the robustness of the synthesized results.	Pages 17-18, Figure 2b, Supplementary Figure 1
Reporting biases	21	Present assessments of risk of bias due to missing results (arising from reporting biases) for each synthesis assessed.	Page 18, Supplementary Figure 2, Supplementary Figure 3
Certainty of evidence	22	Present assessments of certainty (or confidence) in the body of evidence for each outcome assessed.	Pages 20-21, Table 4
**DISCUSSION**			
Discussion	23a	Provide a general interpretation of the results in the context of other evidence.	Pages 22-24
	23b	Discuss any limitations of the evidence included in the review.	Pages 23-24
	23c	Discuss any limitations of the review processes used.	Page 24
	23d	Discuss implications of the results for practice, policy, and future research.	Pages 23.24

Supplementary Table 2 *Cont.*

Section and Topic	Item #	Checklist item	Location where item is reported
**OTHER INFORMATION**			
Registration and protocol	24a	Provide registration information for the review, including register name and registration number, or state that the review was not registered.	Page 4
	24b	Indicate where the review protocol can be accessed, or state that a protocol was not prepared.	Page 4
	24c	Describe and explain any amendments to information provided at registration or in the protocol.	-
Support	25	Describe sources of financial or non-financial support for the review, and the role of the funders or sponsors in the review.	Page 32
Competing interests	26	Declare any competing interests of review authors.	Page 1
Availability of data, code and other materials	27	Report which of the following are publicly available and where they can be found: template data collection forms; data extracted from included studies; data used for all analyses; analytic code; any other materials used in the review.	-

Supplementary Table 3 PRISMA Checklist for Abtracts

Section and Topic	Item #	Checklist item	Reported (Yes/No)
**TITLE**			
Title	1	Identify the report as a systematic review.	Yes
**BACKGROUND**			
Objectives	2	Provide an explicit statement of the main objective(s) or question(s) the review addresses.	Yes
**METHODS**			
Eligibility criteria	3	Specify the inclusion and exclusion criteria for the review.	Yes
Information sources	4	Specify the information sources (e.g. databases, registers) used to identify studies and the date when each was last searched.	Yes
Risk of bias	5	Specify the methods used to assess risk of bias in the included studies.	Yes
Synthesis of results	6	Specify the methods used to present and synthesise results.	Yes
**RESULTS**			
Included studies	7	Give the total number of included studies and participants and summarise relevant characteristics of studies.	Yes
Synthesis of results	8	Present results for main outcomes, preferably indicating the number of included studies and participants for each. If meta-analysis was done, report the summary estimate and confidence/credible interval. If comparing groups, indicate the direction of the effect (i.e. which group is favoured).	Yes
**DISCUSSION**			
Limitations of evidence	9	Provide a brief summary of the limitations of the evidence included in the review (e.g. study risk of bias, inconsistency and imprecision).	Yes
Interpretation	10	Provide a general interpretation of the results and important implications.	Yes
**OTHER**			
Funding	11	Specify the primary source of funding for the review.	No
Registration	12	Provide the register name and registration number.	No
